# Symptoms, signs, and tests: The general practitioner's comprehensive approach towards a cancer diagnosis

**DOI:** 10.3109/02813432.2015.1067512

**Published:** 2015-07

**Authors:** Benedicte Iversen Scheel, Knut Holtedahl

**Affiliations:** Department of Community Medicine, UiT The Arctic University of Norway, Tromsø, Norway

**Keywords:** Early detection of cancer, early diagnosis, family practice, general practice, neoplasms, Norway, pathological conditions, signs and symptoms

## Abstract

*Objective*. To study the relative importance of different tools a GP can use during the diagnostic process towards cancer detection. *Design*. Retrospective cohort study with prospective registration of cancer in general practice. *Setting and subjects*. One hundred and fifty-seven Norwegian general practitioners (GPs) reported 261 cancer patients. *Method*. During 10 consecutive days, GPs registered all patient consultations and recorded any presence of seven focal symptoms and three general symptoms, commonly considered as warning signs of cancer (WSC). Follow-up was done six to 11 months later. For each patient with new or recurrent cancer, the GP completed a questionnaire with medical-record-based information concerning the diagnostic procedure. *Results*. In 78% of cancer cases, symptoms, signs, or tests helped diagnose cancer. In 90 cases, there were 131 consultation-recorded WSC that seemed related to the cancer. Further symptoms were reported for another 74 cases. Different clinical signs were noted in 41 patients, 16 of whom had no previous recording of symptoms. Supplementary tests added information in 59 cases; in 25 of these there were no recordings of symptoms or signs. Sensitivity of any cancer-relevant symptom or clinical finding ranged from 100% for patients with uterine body cancer to 57% for patients with renal cancer. *Conclusion*. WSC had a major role as initiator of a cancer diagnostic procedure. Low-risk-but-not-no-risk symptoms also played an important role, and in 7% of patients they were the only symptoms. Clinical findings and/or supplementary procedures were sometimes decisive for rapid referral.

- Most cancers are symptomatic before diagnosis, but the role of lower risk symptoms and of clinical findings potentially available in general practice is unclear.
- In 164 (62%) of 263 cancer cases, the GPs reported symptoms that helped diagnose cancer. The percentage rose to 78% when clinical findings and test results were added.
- Lower risk symptoms were reported in 31 (12%) patients, and lower risk symptoms without any warning sign in 19 (7%) patients.
- Among patients where clinical signs or tests contributed to diagnosis, symptoms were absent in 39% and in 42%, respectively, showing the necessity of complementing reported symptoms with examinations and tests.

## Background

A general practitioner (GP) can contribute to the early diagnosis of cancer through thoughtful and rational clinical work and referral to more specialized services. For a GP, diagnostic thinking generally starts with symptoms a patient presents. Studies of symptoms and where they lead may produce useful evidence, but more comprehensive studies of how the GP works clinically may increase our understanding. A previous study in general practice showed potential for improvement of medical history-taking, performance of clinical examinations, and choice of supplementary tests and referrals [1].

In a cohort of patients consulting in general practice, it has been shown that GPs manage to distinguish reasonably well between cancer and not-cancer [2]. Another article based on this material reported the frequency and predictive value of warning signs of cancer (WSC) at the time of the consultation, and it was shown that 40% of the cancer patients had presented one or more WSC weeks or months before the diagnosis of cancer had been made [3]. Although several WSC have been studied in different settings [4], less is known about the association between pre-diagnostic cancer and lower risk symptoms, also called low-risk-but-not-no-risk-symptoms, meaning symptoms not ordinarily listed as alarm symptoms of cancer [5]. The relative importance of pre-diagnostic cancer-related clinical findings is not well known, nor is the role of laboratory tests and imaging. In this third cohort-based article we report our findings from the follow-up questionnaire that was designed to collect information on all kinds of symptoms, clinical findings, and supplementary tests that might have triggered a GP's suspicion of cancer after the initial consultation, but before diagnosis.

## Material and methods

All Norwegian GPs (3910) received a questionnaire where they were asked to perform an initial symptom registration during 10 working days for all consecutively consulting patients (Supplementary Appendix 1 available online at http://dx.doi.org/10.3109/02813432.2015.1067512). Completed registrations for 51 073 consulting patients were returned by 396 GPs (10%). Details regarding the initial registrations and exclusions have been described previously [3]. During follow-up 6–7 months later, 283 GPs (71%) reported whether or not any of their original patients had developed cancer. Cases were reported by 157 GPs, who completed a separate questionnaire for each of 261 cancer patients’ illness career (Supplementary Appendix 2 available online at http://dx.doi.org/10.3109/02813432.2015.1067512). New cases of cancer and new recurrences were to be reported, but not previously known cases with stable or progressive disease after initial cancer treatment. The GP was asked to find relevant patient information in the electronic medical record, with details of the patient's present status and the localisation and spread of the tumour. Free text space encouraged comments clarifying the role of symptoms and signs and their possible relationship with the cancer diagnosed.

Sex, date of birth, and date of the initial consultation linked the two registrations. In this way we received medical record-based information both concerning the WSC previously recorded and regarding further symptoms occurring before diagnosis. Various clinical signs and test results were also reported.

The information from the follow-up questionnaire was combined with the initial WSC data, where seven focal and three general WSC were recorded (Table I, with abbreviations used in the article). The WSC studied here have been used in different combinations in several previous studies, and in information campaigns by cancer societies [1,4,6]. More recently, interest has increased concerning lower risk symptoms [7], in this case defined as any non-WSC symptom described by the GPs.

**Table I.** Warning signs of cancer.

	Symptoms studied	Abbreviations
Focal symptoms:	Non-healing skin lesion	Skin lesion
	Lump/nodule	Lump
	Unusual bleeding	Bleeding
	Pigmented skin lesion	Mole
	Persistent digestive problem	Digestive problem
	Cough/hoarseness of uncertain origin	Cough
	Other symptom suspicious of cancer	Other
General symptoms:	Unintentional weight loss	Weight loss
	Unusual fatigue	Fatigue
	Unusual pain	Pain

In order to get the best possible picture of symptoms that could represent cues to the diagnosis of individual cancers, we decided to exclude consultation-recorded, focal WSC that by author consensus had no apparent relationship to the reported type of cancer. The criterion for exclusion of a WSC was that there was no apparent connection between that patient's type of cancer and the symptom. “Other” symptoms were excluded unless a further description made it probable that the symptom was produced by the cancer. In some cases, a focal WSC could be excluded because of a clear relationship with a specified co-morbid condition. Some cases with apparently unrelated symptoms could be related to reported metastatic manifestations at the time of diagnosis.

A minimal number of similar cancers is necessary to do a more detailed analysis of subgroups, i.e. located in one organ or group of organs where one might expect relatively similar symptoms within that group. Seven major types of cancer occurred in more than 20 patients, and three other types of cancer had at least seven patients. We decided to merge cancers with less than seven cases in a “miscellaneous” group, in order to get an idea about symptoms, signs, and tests contributing to the diagnosis of rarer cancers.

### Statistics

All data were analysed in SPSS^™^, version 19 (IBM Corp, Armonk, NY, USA). Chi-square was used to analyse differences between groups. Proportions were calculated with 95% confidence interval (CI). The level of significance was p < 0.05.

## Results

Of the 261 cancer patients, two had a double diagnosis, giving 263 cases. One hundred and six patients had an initial registration of one or more WSC [3]. Of 153 consultation-recorded WSC, 22 WSC were excluded. Ninety of the 106 patients had the remaining 131 WSC (86%) that were considered related to the cancer. Of the 22 WSC considered not related to cancer, seven were apparently related to a specified co-morbid condition. For the 15 others there was no logical link with cancer and further information was insufficient. The sex distribution among the different cancer patients in our study was not significantly different from the sex distribution among Norwegian cancer patients [8]. Mean age ranged from 63 years in breast cancer patients to 76 years in patients with bladder cancer.

Fifty-seven cancer cases (22%) were recurrences. The proportion of recurrent cases varied considerably and was highest for bladder cancer (47%), renal cancer (43%), and breast cancer (34%). There were no recurrences of lung cancer.

Metastases were demonstrated at the time of diagnosis in 31 patients with new cancer (15%) and 21 with recurrent cancer (37%) (p < 0.001). The proportion of metastatic cases was highest for ovarian, pancreatic, and lung cancer. Symptoms from metastases may be expected to be less linked to the organ of the primary tumour, and this seemed to be the case for some patients. More dramatic clinical conditions, such as major infections and neurological symptoms leading to emergency hospitalization, often were due to a cancer that had metastasized.

Table II shows cancer-relevant symptoms and signs and test results reported by the GPs from the medical journal as well as from the consultation, for each type of cancer. In addition to the 90 cases with 131 consultation-recorded WSC, 74 cases had additional symptoms before diagnosis, giving a total of 164 (62%) cases with symptoms. In 22 of the 90 cases with initial WSC, new symptoms added to the consultation-recorded WSC, giving 96 patients with additional symptoms. Of these 96 patients, 31 had lower risk symptoms. Altogether, the 96 patients had 74 symptoms corresponding to WSC and 33 lower risk symptoms. Several non-WSC or lower risk symptoms were reported for most types of cancer. The most frequent of these were six cases with prostatism in prostatic cancer, and dyspnoea was reported in six patients with different cancers. Lower risk symptoms only without any simultaneous WSC were reported for 19 patients (7%) with different kinds of cancer.

**Table II.** Pre-diagnostic, cancer relevant symptom and sign information available for the general practitioner in 261 patients with 263 cases of cancer: Warning signs (WSC) in different types of cancer, along with addititional symptoms, clinical findings and test results that contributed to the diagnosis of cancer.

Type of cancer	N	Initial WSC, considered related to the cancer *	N	Additional symptoms**	N	Clinical signs	N	Supplementary tests	N
Colorectal cancer***	46	Bleeding (5), Digestive problem (10),	18	Digestive problem (5), Bleeding (2), Fatigue (2), Dyspnoea (2), Urinary symptoms (2), Paleness (1), Recurrent pneumonia (1), Leg oedema (1), Rectal symptoms (1), Loss of appetite (1), Mental confusion (1)	16	Poor general condition (4), Tumour at proctoscopy (2) Palpable abdominal tumour (1), Fistula (1), Ascites (1)	9	Anaemia (8), OBS (3), Increased SR (1), Increased CEA (1)	10
Other digestive organs****	22	Bleeding (1), Digestive problem (8), Other (3)	10	Digestive problem (2), Pain (1) Icterus (2), Cholecystitis (1)	6	Palpable supraclavicular tumour (1),	3	Anaemia (1), OBS (1), Incr CRP (1), Incr liver enzymes (1)	4
Lung cancer	23	Digestive problem (3), Cough (4), Other (1)	10	Cough (4), Bleeding (1), Weight loss (2), Pain (3), Dyspnoea (2), Respiratory infection (1), Neurological symptoms (1)	11	COPD (2), Poor general condition (2), Apoplexia (1),	7	X-ray based on symptoms (5), Anaemia (1), Incr SR (2)	8
Skin cancer¤	24	Skin lesion (2), Lump (3), Mole (4), Digestive problem (1), Fatigue (1), Pain (1)	9	Skin lesion (6), Mole (5), Cough (1), Pain (1)	12		0		0
Breast cancer	35	Lump (3), Cough (1), Other (3), Fatigue (2), Pain (1)	9	Lump (7), Cough (1), Pain (2)	10	Retracted mamilla (1), Lung infection (1), Pleural effusion (2)	3	Positive mammography (10)	10
Uterine body cancer	7	Bleeding (2), Digestive problem (1), Other (1), Pain (1)	3	Bleeding (4), Leg oedema (DVT) (1)	5		0		0
Prostate cancer	27	Other (3), Pain (1)	4	Fatigue (1), Pain (1), Prostatism (6), Dyspnoea (1)	8	Lump prostate (4), Enlarged prostate (2), Hard prostate (1), Sepsis (1), Poor general condition (1), Skeletal metastasis (2)	10	Increased PSA (13), Increased SR (1)	14
Renal cancer	7	Other (2)	2	Digestive problem (1), Bleeding (1), Dyspnoea (1)	4		0	Increased SR (1)	1
Bladder cancer	15	Bleeding (4), Cough (1), Weight loss (1)	7	Bleeding (3), Fatigue (1), Urge (1), Urinary retention (1)	5	Nystagmus (1)	1	Anaemia (2), Pos imaging of bladder (1), Pos. Urinary cytology (1)	4
Lymphoid/hematopoietic cancer¤¤	28	Lump (2), Bleeding (1), Digestive problem (1)	10	Lump (3), Digestive problem (2)	8	Bacterial infections: Sepsis (1)	3	Leukocytosis (2), Increased SR (2)	6
Miscellaneous cases¤¤¤	29	Lump (2), Bleeding (1), Digestive problem (3), Cough (1), Weight loss (1), Fatigue (3), Pain (2)	8	Lump (5), Pain (4), Mental confusion (2), Diplopia (1)	11	Tumour on cervix uteri (1), Sialolthiasis (1), Unilateral elevation of floor of mouth (1), Tumour lip (1), Tumour oral mucosa (1), Tumour tongue (1)	5	Increased CA125 (1), Anaemia (1), OBS (1)	2
All types of cancer	263		90		96		41		59

N = Number of cases of cancer.

OBS = Occult blood in stool, SR = Sedimentation Rate, CEA = Carcino-Embryonal Antigen, CA125 = Cancer antigen 125, Incr = Increased, COPD = Chronic obstructive pulmonary disease.

*The 22 excluded WSC were: ‘Skin lesion’ 3 (colorectal 2, prostate 1), ‘Lump’ 2 (prostate), ‘Bleeding’ 3 (breast 2, skin 1), ‘Digestive problem’1 (skin), ‘Cough’ 5 (colorectal 1, other digestive 1, skin 2, prostate 1), ‘Other’ 6 (colorectal 1, prostate 3, skin 1, ovary 1), ‘Weight loss’ 1 (skin), ‘Pain’ 1, (skin).

**Additional symptoms include non-WSC symptoms and WSC not recorded at the initial consultation. Symptoms described in the medical journal after initial registration of WSC. Because of multiple symptoms and/or signs in some patients, the sum of symptoms listed in the columns may exceed N (number of cases).

***Colorectal: 1 Appendix, 3 coecum, 5 ascendum, 1 transversum, 5 sigmoid, 19 colon not specified, 12 rectum.

****Other digestive organs: 3 oesophageal cancer, 3 stomach cancer, 2 neuroendocrine cancer (carcinoid) of small intestine, 1 hepatocellular carcinoma, 2 biliary cancer, 1 cancer of papilla Vaterii, 9 pancreatic cancer.

¤Skin cancer: 12 malignant melanoma, 12 squamous cell carcinoma.

¤¤Lymphoid/hematopoietic cancer: 2 Mb Hodgkin, 7 Non-Hodgkin lymphoma, 3 lymphoma not spec (2 of these in stomach), 6 myleomatosis/myelodysplastic syndrome, 10 leukemia (3 AML, 3 CML, 3 CLL, 1 leukemia not specified).

¤¤¤6 cancer of mouth, pharynx, 2 laryngeal cancer, 1 mesothelioma, 4 soft tissue cancer, 4 cervical cancer, 3 ovarian cancer, 3 testicular cancer, 3 glioblastomas, 2 thyroid cancer, 2 cancer of unknown origin.

Symptoms considered as typical WSC for some common cancers were not necessarily very frequent. “Digestive problem” was noted in 25 cases and “bleeding” in eight cases of the 68 cancers of the digestive organs. Of the 35 breast cancer patients, ten had a “lump”. Of 23 lung cancer patients, eight had “cough”. In 12 cases of malignant melanoma “mole” was noted in seven cases, “lump”, in two cases and “skin lesion” in two cases. The last case lacked information on how it was diagnosed. This kind of varying symptom perception was also found for the 12 cases of squamous cell carcinoma of skin, although “skin lesion” had been noted in half of the cases.

Clinical findings were noted in 41 patients (16%), 16 (39%) of whom had no previous recording of symptoms, and these findings varied considerably. Inspection and palpation including rectal palpation often gave clues to the diagnosis. Supplementary tests added information in 59 cases (22%); in 25 (42%) of these there were no recordings of symptoms or signs. Among test-based signals noted by the GP, anaemia was clearly the most frequent and occurred in 15 patients. Of eight patients with anaemia and colorectal cancer, only one patient also had a recording of “bleeding”. Of the six patients with anaemia and other cancers, two also had “bleeding”. Compared with the 25 patients in total with a recording of “ bleeding” either at the initially recorded consultation or later on before diagnosis, anaemia was more than half as frequent as an independent signal of cancer. Occult blood in stool was noted in only three cases of colorectal cancer, in one case of stomach cancer, and in one generalized cancer (see Table II).

Table III reflects how GPs can gain progressively more comprehensive diagnostic knowledge concerning a patient's ailment. In 78% (95% CI 73–83%) of the cancer cases, the GPs reported symptoms, signs, or tests that helped diagnose cancer (Table III). Sensitivity of any cancer-relevant symptom or clinical finding ranged from 100% for patients with uterine body cancer to 57% for patients with renal cancer. Sensitivity of any cancer-related symptom was 62% and almost twice as high as the sensitivity of WSC recorded when the patient consulted. Screening procedures like mammography, PSA, or cervical smear had been performed on several patients, and among these 11 (4%) of 263 cases were specified by the GP as asymptomatic. In the 58 cases not appearing in Table III, the diagnosis was either made during investigation for other disease, or there was a lack of information regarding symptoms and signs. However, in about half of these cases the GP had noted that the diagnostic process had been initiated on the basis of symptoms that were not specified.

**Table III.** Sensitivity of cancer relevant symptoms and signs in relation to different types of cancer. 261 patients with 263 cases of cancer (N).

		Consultation-recorded WSC	Any pre-diagnostic symptom	Symptoms and clinical findings	Symptoms, signs and test results
Type of cancer	N	N	Sensitivity	95% CI	N	Sensitivity	95% CI	N	Sensitivity	95% CI	N	Sensitivity	95% CI
Colorectal	46	18	39%	26–54%	30	65%	51–77%	33	72%	57–83%	37	80%	67–89%
Other digestive organs	22	10	45%	27–65%	15	68%	47–84%	16	73%	52–87%	17	77%	57–90%
Lung	23	10	43%	26–63%	17	74%	54–87%	20	87%	68–95%	21	91%	73–98%
Skin	24	9	38%	21–57%	21	88%	69–96%	21	88%	69–96%	21	88%	69–96%
Breast	35	9	26%	14–42%	15	43%	28–59%	17	49%	33–64%	26	74%	58–86%
Uterine body	7	3	43%	16–75%	7	100%	65–100%	7	100%	65–100%	7	100%	65–100%
Prostate	27	4	15%	6–32%	11	41%	25–59%	14	52%	34–69%	18	67%	48–81%
Renal	7	2	29%	8–64%	4	57%	25–84%	4	57%	25–84%	4	57%	25–84%
Bladder	15	7	47%	25–70%	11	73%	48–89%	11	73%	48–89%	13	87%	62–96%
Lymphoid/hematopoietic	28	10	36%	21–54%	15	54%	36–70%	17	61%	42–76%	20	71%	53–85%
Miscellaneous	29	8	28%	15–46%	18	62%	44–77%	20	69%	51–83%	21	72%	54–85%
All types of cancer	263	90	34%	29–40%	164	62%	56–68%	180	68%	63–74%	205	78%	73–83%

WSC = Warning signs of cancer.

Any pre-diagnostic symptom = WSC + additional (non-WSC) symptoms. Information from consultation registrations and from medical records combined.

N for Any pre-diagnostic symptom may be lower than the sum of cases with symptoms in Table II, because one patient may have both initial and (different) additional symptoms.

N for clinical findings adds only cases where no symptoms were recorded, and N that includes test results adds cases where there was no recorded contributions from symptoms or clinical findings.

## Discussion

### Statement of principal findings

The intention of this paper is to describe symptoms, signs, and tests that contributed to the diagnosis of cancer after the patients had consulted in general practice. Approximately one in three patients presented a WSC during the consultation and almost two in three experienced a cancer-relevant symptom before diagnosis. These figures testify to the importance of well-known alarm symptoms and to the variety of lower risk symptoms that also may signal cancer. Seven per cent of patients experienced lower risk symptoms only, while the diagnostic contribution from lower risk symptoms seemed modest when there was also a WSC. The above figures are minimum figures because some patients had unspecified symptoms, and because there may have been symptoms not reported or described in the medical journal. If one includes clinical examination and results from simple tests that are accessible for most GPs, symptoms and/or signs were present in almost four of five patients.

Among the remaining cases, some were asymptomatic and were detected through screening or case finding. At least seven of 10 positive mammograms were routine screening cases, contributing to the low symptom sensitivity figures for breast cancer. However, the rationale behind screening procedures is the possibility of diagnosing a cancer before symptoms appear, because local symptoms for some cancers are associated with systemic disease. High sensitivity of symptoms is valuable mainly when symptoms tend to appear early, like haematuria in bladder cancer or bleeding from a uterine body cancer. Most of the PSA tests were ordered on the basis of symptoms, but in some cases this was not clear. The main picture is that in most cases of cancer there will be manifestations of the disease that are potentially detectable by the GP. Clinical signs sometimes gave cues to increased suspicion and appropriate referral. These were important in the few cases of oral cancer. In renal cancer cases neither symptoms nor signs were prominent.

Among useful laboratory results, anaemia is yet again shown to merit an explanation when diagnosed. GPs should perform a haemoglobin measurement in unclear cases, whether or not “bleeding” is present. Occult blood in stool (OBS) may have greater diagnostic utility than has been shown in our study, and symptoms plus a positive test warrant further investigation [9].

The probability of diagnosing a recurrent cancer was greater for some forms of cancer, i.e. urinary cancer and breast cancer. That there were no recurrences of lung cancer reflects the serious nature of this type of cancer. It is encouraging that only one in 10 new cancers had evidence of metastatic spread at the time of diagnosis. This means that competent cancer-diagnostic work by the GP may represent a prognostic difference for many patients. The task is even more challenging for recurrent cancer cases, where one in three patients had metastases when diagnosed. A previous cancer diagnosis is always a red flag for GPs.

### Strengths and weaknesses of our study

The prospective nature of the follow-up ascertained that neither the patient nor the GP knew about the cancer diagnosis at the time of WSC registration [10]. However, symptoms presenting before diagnosis but after the initial consultation do not show in our cross-sectional consultation data. The combination with medical record-based symptom information from the GP allowed for a more complete picture of the diversity of symptoms preceding a diagnosis of cancer. This picture is comparable with the spectrum of symptoms found in case-control interviews or questionnaire studies, but without important potential sources of bias like recall bias, which may be different for a personally affected patient and a more neutral control patient. Data in medical records are imperfect, but they offer a unique opportunity to review an entire clinical course [11]. Because the study dealt with cancer, it is possible that any under-reporting may have been more important for lower-risk symptoms than for WSC.

The distinction between additional symptoms and clinical signs, or between signs and supplementary tests, was not always clear, as in the cases of urinary retention and of leg oedema due to deep vein thrombosis, or in anaemia. The important point is that such symptoms, signs, or test results offer diagnostic possibilities for the GP.

The symptoms cover a period of up to 11 months preceding the diagnoses, although most recorded symptoms occurred during the last three to four months before diagnosis. With a few exceptions, most cancer-related symptoms seem to have been recorded, forming a relatively complete picture of the broad variation in symptoms for each type of cancer. Despite the modest number of cancer cases, it is probable that the spectrum of symptoms resembles the spectrum for all cases of similar cancers in Norway. This is because all consulting patients were registered consecutively, and the cancer cases can be assumed to have been distributed randomly among the GPs. The low response rate among GPs was foreseen and not considered important because of this haphazard distribution of cancer cases in the surgeries.

The number of cancer cases of each type is small, with broad confidence intervals for calculated sensitivity figures. This limits the possibility of finding nuances in the pre-diagnostic role of symptoms, clinical procedures, etc. However, where differences between types of cancer could be expected, findings mainly go in the expected direction, suggesting that the data are reliable.

### Comparison with other studies

We think our study gives a rather comprehensive picture of the information available to the GP from consultation to diagnosis, and more so than most other studies. It is established that alarm symptoms are valid in relation to cancer and in many cases contribute to the diagnosis [6]. However, the absence of such symptoms does not mean absence of cancer [12]. Hamilton [5] emphasized the important role of lower risk symptoms, which are less apt to be referred to “fast track” diagnosis. In primary care, variability of symptoms has been demonstrated for colorectal cancer [13–16], bladder cancer [17], uterine cancer [18], and pancreatic cancer [19]. For urological cancer it has been found that the presence of other symptoms in addition to haematuria did not influence predictive value [20]. Anaemia has been studied, especially in relation to colorectal cancer [21]. The conclusions in these studies are not very different from ours. Prostatism is a problematic symptom because it signals both benign and malignant growth, and prostatic cancer cells are very common in elderly men. When a PSA test is considered, patients should be informed about the nature and possible consequences of PSA test results [22,23].

### Implications for clinical practice and further research

Our study improves our understanding of the clinical road towards a cancer diagnosis in general practice. It adds to the understanding of how a GP can deal with the rather unspecific symptoms that patients present daily, where cancer is one possibility among many others. WSC must be explained, and even lower-risk symptoms cannot be overlooked. Our findings show how the patient collection of further information through appropriate clinical examination and supplementary testing can provide a more rational basis for referral. It is important to seek combinations of symptoms and signs and perhaps even “gut feeling” [24,25], where the combination maximizes sensitivity [26] as well as specificity, in order to increase the positive predictive value. Commonly, it is the combined diagnostic approach that allows the distinction of probable cancer from non-cancer [2]. Some of the clinical findings reported in our patients may have been decisive for a rapid referral to specialist diagnostics. Cancers with few symptoms and signs like renal cancer, or with symptoms and signs usually associated with advanced disease, like pancreatic or ovarian cancer, should be consciously considered by the GP when there are vague but persistent symptoms. Some types of cancer had more distinct symptoms than others, but rare cancers seem to have about the same symptom frequency as more frequent cancers.

The cognitive mechanisms that make a GP suspect cancer in a patient are complex [27,28] and deserve attention in medical schools. Errors are unavoidable but may be minimized [29], and the GPs’ closeness to patients is important. The combination of high-frequency symptoms and low-frequency cancers is a challenge to the diagnostic skills of GPs. The diagnostic role of non-WSC symptoms and of clinical findings merits further research.

## Supplementary Material

Supplementary Appendix 1

Supplementary Appendix 2

## References

[C1] Holtedahl KA Diagnosis of cancer in general practice: A study of delay problems and warning signals of cancer, with implications for public cancer information and for cancer diagnostic strategies in general practice. ISM skriftserie nr. 16. Tromsø: University of Tromsø; 1991 http://hdl.handle.net/10037/2325.

[C2] Scheel BI, Ingebrigtsen SG, Thorsen T, Holtedahl K Cancer suspicion in general practice: The role of symptoms and patient characteristics, and their association with subsequent cancer. Br J Gen Pract 2013;63:e627–35. 23998843 10.3399/bjgp13X671614 PMC3750802

[C3] Ingebrigtsen SG, Scheel BI, Hart B, Thorsen T, Holtedahl K Frequency of “warning signs of cancer” in Norwegian general practice, with prospective recording of subsequent cancer. Fam Pract 2013;30:153–60. 23097250 10.1093/fampra/cms065

[C4] Shapley M, Mansell G, Jordan JL, Jordan KP Positive predictive values of >/ = 5% in primary care for cancer: Systematic review. Br J Gen Pract 2010;60:e366–77. 20849687 10.3399/bjgp10X515412 PMC2930247

[C5] Hamilton W The CAPER studies: Five case-control studies aimed at identifying and quantifying the risk of cancer in symptomatic primary care patients. Br J Cancer 2009;101:S80–S6. 19956169 10.1038/sj.bjc.6605396 PMC2790706

[C6] Jones R, Latinovic R, Charlton J, Gulliford MC Alarm symptoms in early diagnosis of cancer in primary care: Cohort study using General Practice Research Database. BMJ 2007;334:1040. 17493982 10.1136/bmj.39171.637106.AE PMC1871798

[C7] Hamilton W Cancer diagnosis in primary care. Br J Gen Pract 2010;60:121–8. 20132704 10.3399/bjgp10X483175 PMC2814263

[C8] Cancer Registry of Norway Cancer in Norway 2011: Cancer incidence, mortality, survival and prevalence in Norway. Oslo: Cancer Registry of Norway; 2013.

[C9] Holtedahl KA Probability revision in general practice: The cases of occult blood in stool in patients with indigestion, and daily smoking in patients who cough. Allgemeinmedizin 1990;19:35–8. http://www2.uit.no/ikbViewer/Content/237422/Probability.pdf

[C10] Sedgwick P Retrospective cohort studies: Advantages and disadvantages. BMJ. 2014;348:g1072. 10.1136/bmj.g2979 25134102

[C11] Feinstein AR, Pritchett JA, Schimpff CR The epidemiology of cancer therapy, IV: The extraction of data from medical records. Ann Intern Med 1969;123:571–90. 5780702

[C12] Wauters H, Van Casteren V, Buntinx F Rectal bleeding and colorectal cancer in general practice: Diagnostic study. BMJ 2000;321:998–9. 11039968 10.1136/bmj.321.7267.998 PMC27509

[C13] Barrett J, Jiwa M, Rose P, Hamilton W Pathways to the diagnosis of colorectal cancer: An observational study in three UK cities. Fam Pract 2006;23:15–19. 16286462 10.1093/fampra/cmi093

[C14] Bekkink MO, McCowan C, Falk GA, Teljeur C, Van de Laar FA, Fahey T Diagnostic accuracy systematic review of rectal bleeding in combination with other symptoms, signs and tests in relation to colorectal cancer. Br J Cancer 2010;102:48–58. 19935790 10.1038/sj.bjc.6605426 PMC2813743

[C15] Hamilton W, Lancashire R, Sharp D, Peters TJ, Cheng KK, Marshall T The risk of colorectal cancer with symptoms at different ages and between the sexes: A case-control study. BMC Medicine 2009;7:17. 19374736 10.1186/1741-7015-7-17 PMC2675533

[C16] Ford AC, Veldhuyzen van Zanten SJ, Rodgers CC, Talley NJ, Vakil NB, Moayyedi P Diagnostic utility of alarm features for colorectal cancer: Systematic review and meta-analysis. Gut 2008;57:1545–53. 18676420 10.1136/gut.2008.159723

[C17] Shephard EA, Stapley S, Neal RD, Rose P, Walter FM, Hamilton WT Clinical features of bladder cancer in primary care. Br J Gen Pract 2012;62:e598–e604. 22947580 10.3399/bjgp12X654560 PMC3426598

[C18] Walker S, Hyde C, Hamilton W Risk of uterine cancer in symptomatic women in primary care: Case-control study using electronic records. Br J Gen Pract 2013;63: e643–e8. 23998845 10.3399/bjgp13X671632 PMC3750804

[C19] Collins GS, Altman DG Identifying patients with undetected pancreatic cancer in primary care: An independent and external validation of QCancer^®^ (Pancreas). Br J Gen Pract 2013;63:e636–e42. 23998844 10.3399/bjgp13X671623 PMC3750803

[C20] Bruyninckx R, Buntinx F, Aertgeerts B, Van Casteren V The diagnostic value of macroscopic haematuria for the diagnosis of urological cancer in general practice. Br J Gen Pract 2003;53:31–5. 12564274 PMC1314489

[C21] Hamilton W, Lancashire R, Sharp D, Peters TJ, Cheng KK, Marshall T The importance of anaemia in diagnosing colorectal cancer: A case-control study using electronic primary care records. Br J Cancer 2008;98:323–7. 18219289 10.1038/sj.bjc.6604165 PMC2361444

[C22] Wilt TJ, Ahmed HU Prostate cancer screening and the management of clinically localized disease. BMJ 2013;346: f325. 23360718 10.1136/bmj.f325 PMC3938276

[C23] Graham J, Kirkbride P, Cann K, Hasler E, Prettyjohns M Prostate cancer: Summary of updated NICE guidance. BMJ 2014;348:f7524. 24401467 10.1136/bmj.f7524

[C24] Buntinx F, Mant D, Van den Bruel A, Donner-Banzhof N, Dinant GJ Dealing with low-incidence serious diseases in general practice. Br J Gen Pract 2011;61:43–6. 21401991 10.3399/bjgp11X548974 PMC3020049

[C25] Stolper E, Wiel M, Royen P, Bokhoven M, Weijden T, Dinant G Gut feelings as a third track in general practitioners’ diagnostic reasoning. J Gen Int Med 2011;26:197–203. 10.1007/s11606-010-1524-5 PMC3019314 20967509

[C26] Rubin G, Hamilton W Alarm features of colorectal cancer. Gut 2009;58:1026. 19520894

[C27] Johansen M-L, Holtedahl KA, Rudebeck CE How does the thought of cancer arise in a general practice consultation? Interviews with GPs. Scand J Prim Health Care 2012;30:135–40. 22747066 10.3109/02813432.2012.688701 PMC3443936

[C28] Kostopoulou O, Delaney BC, Munro CW Diagnostic difficulty and error in primary care: A systematic review. Fam Pract 2008;25:400–13. 18842618 10.1093/fampra/cmn071

[C29] Singh H, Giardina T, Meyer AD, Forjuoh SN, Reis MD, Thomas EJ Types and origins of diagnostic errors in primary care settings. JAMA Intern Med 2013;173:418–25. 23440149 10.1001/jamainternmed.2013.2777 PMC3690001

