# Supplementary material for: Symptoms, signs, and tests: The general practitioner's comprehensive approach towards a cancer diagnosis
Source: Scand J Prim Health Care. 2015 Jul;33(3):170–7. doi: 10.3109/02813432.2015.1067512 (PMC4750720; doi:10.3109/02813432.2015.1067512)
Supplement: Supplementary Appendix 1 [file ipri-33-151S1.pdf]

Supplementary material for Scheel BI & Holtedahl K. Symptoms, signs, and tests: The general practitioner's comprehensive approach towards a cancer diagnosis, Scandinavian Journal of Primary Health Care, 2015, doi: 10.3109/02813432.2015.1067512

Supplementary Appendix 1

SYMPTOMS AND CANCER

GP number: | 01

Question 11: *Number of weeks*
Question 12–13 and 27–28:
Only one X.
All other groups:
Several X's possible

DATE

	SEX		DATE OF BIRTH			FOCAL SYMPTOM							GENERAL SYMPTOM			TIME			ORGAN SYSTEM									ACTION			Other illness	CANCER		PAST	NOTES OR COMMENTS
						1	2	3	4	5	6	7	8	9	10	11	12	13	14	15	16	17	18	19	20	21	22	23	24	25	26	27	28	29	
	♂	♀	DD	MM	YY	Soare which does not heal	Lump	Unusual bleeding	Mole	Persistent digestive problem	Nagging cough / hoarseness	Other cancer suspect symptom	Unintentional weight loss	Unusual fatigue	Unusual pain	Duration of symptoms, in weeks	First consultation	NOT first consultation	Oral cavity / pharynx	Gastrointestinal tract	Airways	Breast	Genitals	Urinary tract	Skin	CNS / Eye	Blood / Lymph	Lab tests, all types	X-Ray / Imaging	Referral / Admission	Significant comorbidity	Cancer not likely	Cancer possible - follow-up needed	Previous cancer	**NB!** Especially question 7, 23–25, 26 and 29
01																																			
02																																			
03																																			
04																																			
05																																			
06																																			
07																																			
08																																			
09																																			
10																																			
11																																			
12																																			
13																																			
14																																			
15																																			
16																																			
17																																			
