# Supplementary material for: Symptoms, signs, and tests: The general practitioner's comprehensive approach towards a cancer diagnosis
Source: Scand J Prim Health Care. 2015 Jul;33(3):170–7. doi: 10.3109/02813432.2015.1067512 (PMC4750720; doi:10.3109/02813432.2015.1067512)
Supplement: Supplementary Appendix 2 [file ipri-33-151S2.pdf]

Supplementary Appendix 2

Kreft og allmennpraksis

SYMPTOMS AND CANCER
(cancer and general practice)

1- Date of filling the questionnaire (ddmmyyyy) 2 0

2- General practioner's code:

3- I have reviewed all medical records for all patients registered during the initial study period:
(fill in) YES

4- Number of patients diagnosed with new cancer between your initial registration period and now:

5- Number of patients diagnosed with recurrence of a previously treated cancer between your initial registration period and now:

If you found any cancers, please fill in one patient form for each cancer patient
If you didn't find any cancer cases (number = 0) go directly to question 46.

PATIENT FORM
6- Patient's date of birth (ddmmyy)

7- Patient's gender (INSCRIBE F OR M)

8- Year of your first contact with this patient (yy)

9- Month and year for confirmed cancer diagnosis (mmyy)

10- Current cancer type: (fill in one cell only)
New cancer:
New recurrence of cancer:

Kreft og allmennpraksis

SYMPTOMS AND CANCER
(cancer and general practice)

1- Date of filling the questionnaire (ddmmyyyy) 2 0

2- General practioner's code:

3- I have reviewed all medical records for all patients registered during the initial study period:
(fill in) YES

4- Number of patients diagnosed with new cancer between your initial registration period and now:

5- Number of patients diagnosed with recurrence of a previously treated cancer between your initial registration period and now:

If you found any cancers, please fill in one patient form for each cancer patient
If you didn't find any cancer cases (number = 0) go directly to question 46.

PATIENT FORM
6- Patient's date of birth (ddmmyy)

7- Patient's gender (INSCRIBE F OR M)

8- Year of your first contact with this patient (yy)

9- Month and year for confirmed cancer diagnosis (mmyy)

10- Current cancer type: (fill in one cell only)
New cancer:
New recurrence of cancer:

Kreft og allmennpraksis

SYMPTOMS AND CANCER

(cancer and general practice)

1- Date of filling the questionnaire (ddmmyyyy) [ ][ ][ ][ ][2][0][ ][ ]

2- General practioner's code:

3- I have reviewed all medical records for all patients registered during the initial study period:
(fill in) YES

4- Number of patients diagnosed with new cancer between your initial registration period and now:

5- Number of patients diagnosed with recurrence of a previously treated cancer between your initial registration period and now:

If you found any cancers, please fill in one patient form for each cancer patient
If you didn't find any cancer cases (number = 0) go directly to question 46.

PATIENT FORM

6- Patient's date of birth (ddmmyy)

7- Patient's gender (INSCRIBE F OR M)

8- Year of your first contact with this patient (yy)

9- Month and year for confirmed cancer diagnosis (mmyy)

10- Current cancer type: (fill in one cell only)
New cancer:
New recurrence of cancer:

Kreft og allmennpraksis

SYMPTOMS AND CANCER

(cancer and general practice)

1- Date of filling the questionnaire (ddmmyyyy) [ ][ ][ ][ ][2][0][ ][ ]

2- General practioner's code:

3- I have reviewed all medical records for all patients registered during the initial study period:
(fill in) YES

4- Number of patients diagnosed with new cancer between your initial registration period and now:

5- Number of patients diagnosed with recurrence of a previously treated cancer between your initial registration period and now:

If you found any cancers, please fill in one patient form for each cancer patient
If you didn't find any cancer cases (number = 0) go directly to question 46.

PATIENT FORM

6- Patient's date of birth (ddmmyy)

7- Patient's gender (INSCRIBE F OR M)

8- Year of your first contact with this patient (yy)

9- Month and year for confirmed cancer diagnosis (mmyy)

10- Current cancer type: (fill in one cell only)
New cancer:
New recurrence of cancer:

Kreft og allmennpraksis

SYMPTOMS AND CANCER

(cancer and general practice)

1- Date of filling the questionnaire (ddmmyyyy) 20

2- General practioner's code:

3- I have reviewed all medical records for all patients registered during the initial study period:
(fill in) YES

4- Number of patients diagnosed with new cancer between your initial registration period and now:

5- Number of patients diagnosed with recurrence of a previously treated cancer between your initial registration period and now:

If you found any cancers, please fill in one patient form for each cancer patient
If you didn't find any cancer cases (number = 0) go directly to question 46.

PATIENT FORM

6- Patient's date of birth (ddmmyy)

7- Patient's gender (INSCRIBE F OR M)

8- Year of your first contact with this patient (yy)

9- Month and year for confirmed cancer diagnosis (mmyy)

10- Current cancer type: (fill in one cell only)
New cancer:
New recurrence of cancer:

Kreft og allmennpraksis

SYMPTOMS AND CANCER

(cancer and general practice)

1- Date of filling the questionnaire (ddmmyyyy) 20

2- General practioner's code:

3- I have reviewed all medical records for all patients registered during the initial study period:
(fill in) YES

4- Number of patients diagnosed with new cancer between your initial registration period and now:

5- Number of patients diagnosed with recurrence of a previously treated cancer between your initial registration period and now:

If you found any cancers, please fill in one patient form for each cancer patient
If you didn't find any cancer cases (number = 0) go directly to question 46.

PATIENT FORM

6- Patient's date of birth (ddmmyy)

7- Patient's gender (INSCRIBE F OR M)

8- Year of your first contact with this patient (yy)

9- Month and year for confirmed cancer diagnosis (mmyy)

10- Current cancer type: (fill in one cell only)
New cancer:
New recurrence of cancer:

Kreft og allmennpraksis

SYMPTOMS AND CANCER

(cancer and general practice)

1- Date of filling the questionnaire (ddmmyyyy) 2 0

2- General practioner's code:

3- I have reviewed all medical records for all patients registered during the initial study period:
(fill in) YES

4- Number of patients diagnosed with new cancer between your initial registration period and now:

5- Number of patients diagnosed with recurrence of a previously treated cancer between your initial registration period and now:

If you found any cancers, please fill in one patient form for each cancer patient
If you didn't find any cancer cases (number = 0) go directly to question 46.

PATIENT FORM

6- Patient's date of birth (ddmmyy)

7- Patient's gender (INSCRIBE F OR M)

8- Year of your first contact with this patient (yy)

9- Month and year for confirmed cancer diagnosis (mmyy)

10- Current cancer type: (fill in one cell only)
New cancer:
New recurrence of cancer:

Kreft og allmennpraksis

SYMPTOMS AND CANCER

(cancer and general practice)

1- Date of filling the questionnaire (ddmmyyyy) 2 0

2- General practioner's code:

3- I have reviewed all medical records for all patients registered during the initial study period:
(fill in) YES

4- Number of patients diagnosed with new cancer between your initial registration period and now:

5- Number of patients diagnosed with recurrence of a previously treated cancer between your initial registration period and now:

If you found any cancers, please fill in one patient form for each cancer patient
If you didn't find any cancer cases (number = 0) go directly to question 46.

PATIENT FORM

6- Patient's date of birth (ddmmyy)

7- Patient's gender (INSCRIBE F OR M)

8- Year of your first contact with this patient (yy)

9- Month and year for confirmed cancer diagnosis (mmyy)

10- Current cancer type: (fill in one cell only)
New cancer:
New recurrence of cancer:
